# When Fiber Does Not Protect: Evaluating Statistical Interaction Between Dietary Fiber, Sodium-to-Potassium Ratio, and Metabolic Syndrome in South Korean Adults

**DOI:** 10.3390/healthcare14193145

**Published:** 2026-09-22

**Authors:** Cihad Dundar

**Affiliations:** Department of Public Health, Faculty of Medicine, Ondokuz Mayıs University, Samsun 55200, Türkiye; cdundar@omu.edu.tr

**Keywords:** dietary sodium-potassium ratio, dietary fiber, metabolic syndrome, hypertension, KNHANES, moderation analysis

## Abstract

**Highlights:**

**What are the main findings?**

- A higher dietary sodium-to-potassium ratio was independently associated with increased odds of hypertension, metabolic syndrome, and greater continuous metabolic syndrome severity among South Korean adults.
- Dietary fiber intake significantly modified the association between the sodium-to-potassium ratio and metabolic syndrome outcomes, showing a statistical interaction rather than a simple protective attenuation.

**What are the implications of the main findings?**

- Dietary recommendations should consider whole food matrices, such as sodium-rich traditional vegetable preparations, rather than assuming fiber intake operates independently of background sodium levels.
- Population-level preventive health strategies in South Korea should prioritize reducing the overall sodium-to-potassium ratio alongside comprehensive dietary patterns, acknowledging that cross-sectional data establish statistical associations rather than causal relationships.

**Abstract:**

**Objectives:** Metabolic syndrome (MetS) represents a major public health concern strongly associated with dietary factors. High sodium and low potassium intakes are established cardiometabolic risk factors, and dietary fiber has been hypothesized to exert a buffering influence. In this cross-sectional study, whether dietary fiber intake moderates the association between the dietary sodium-to-potassium (Na/K) ratio and MetS outcomes was evaluated in South Korean adults. **Methods:** A final unweighted sample of 8354 adults aged 20–59 years from the pooled 2022–2024 Korea National Health and Nutrition Examination Survey (KNHANES) was analyzed, representing a weighted population of 27,293,126 adults. The dietary Na/K ratio and fiber intake were estimated using a single 24 h dietary recall. MetS was defined using modified National Cholesterol Education Program Adult Treatment Panel III criteria alongside a validated continuous MetS severity score. Multivariate survey-weighted regression models were estimated to evaluate associations with metabolic outcomes, with adjustment for age, sex, body mass index, smoking status, alcohol consumption, and total energy intake. Moderation by dietary fiber was assessed using product-interaction terms, simple-slope analyses, and Johnson–Neyman techniques. **Results:** Overall MetS prevalence was 23.0%. A higher Na/K ratio was independently associated with increased odds of hypertension (OR = 1.144, 95% CI: 1.023–1.279, *p* = 0.018) and metabolic syndrome (OR = 1.204, 95% CI: 1.076–1.347, *p* = 0.001), as well as a higher continuous MetS severity score (β = 0.088, 95% CI: 0.034–0.141, *p* = 0.001). Contrary to the hypothesized buffering role, dietary fiber intake demonstrated a statistically significant interaction with the Na/K ratio for metabolic syndrome outcomes (binary MetS interaction: OR = 1.010, 95% CI: 1.002–1.018, *p* = 0.013; severity score interaction: β = 0.0060, 95% CI: 0.0023–0.0096, *p* = 0.001). This interaction was not statistically significant for hypertension (*p* = 0.581). **Conclusions:** Dietary fiber intake demonstrated a positive statistical interaction with the dietary Na/K ratio, contrary to the hypothesized attenuating influence. This observed statistical interaction likely reflects contextual dietary factors, such as overlapping plant sources of sodium and fiber, residual confounding, or reverse causality rather than an inherently adverse biological mechanism. These interaction patterns warrant longitudinal confirmation before informing public health dietary guidance.

## 1. Introduction

In South Korea, hypertension and metabolic syndrome (MetS) rank among the most critical public health challenges, driving much of the national burden of cardiovascular disease, type 2 diabetes, and premature mortality [1]. Excessive dietary sodium is a long-acknowledged, modifiable risk factor for high blood pressure. South Korea records some of the highest average sodium intake levels in the world, driven largely by a food culture rich in salted, fermented, and seasoned items such as kimchi, jjigae (stews), and various banchan (side dishes) [2]. Although national sodium reduction programs have been active since 2012, they have achieved only limited success in pulling population intake down toward WHO-recommended targets [3].

Hypertension prevention has long focused primarily on reducing sodium intake. However, a growing body of evidence indicates that the balance between sodium and potassium offers a physiologically more meaningful indicator of cardiometabolic risk than evaluating either electrolyte in isolation [4]. Potassium offsets key mechanisms through which sodium elevates blood pressure, including renal sodium retention, sympathetic nervous system overactivation, and impaired vascular endothelial function. A diet simultaneously high in sodium and low in potassium can, therefore, confer risks beyond what sodium intake alone would predict [5]. Multiple cohort and cross-sectional studies worldwide have linked an elevated sodium-to-potassium (Na/K) ratio directly to higher blood pressure, hypertension incidence, and cardiovascular disease [6,7], with newer findings showing a parallel association with metabolic syndrome (MetS) and its individual components [8]. In South Korea, Baek and Kim demonstrated this relationship using KNHANES VII (2016–2018) data, showing that a higher dietary Na/K ratio correlated with increased MetS risk among adults [9]. That analysis, however, relied strictly on binary diagnostic criteria for MetS and did not evaluate whether other dietary factors might modify this relationship.

Dietary fiber has received substantial attention as a potential safeguard against cardiometabolic disease. Proposed physiological mechanisms include delayed gastric emptying and blunted glucose absorption, enhanced insulin sensitivity, favorable modulation of gut microbiota, increased short-chain fatty acid production (SCFA), and improved lipid profiles [10], alongside microbiome-mediated blood pressure r regulation driven by SCFA signaling [11]. These pathways support the hypothesis that adequate dietary fiber intake might buffer the cardiometabolic burden associated with an unfavorable Na/K ratio, such as by strengthening vascular and metabolic resilience to sodium-related stress. To our knowledge, however, this moderating hypothesis has not been formally tested in a nationally representative Korean population using an interaction-based statistical framework.

Most existing studies on MetS risk rely exclusively on binary diagnostic criteria. While clinically intuitive, these criteria fail to capture continuous variation in the severity of metabolic risk, potentially reducing sensitivity when detecting statistical moderation [8,9,12]. Continuous, population-specific severity scores, derived through confirmatory factor analysis and validated separately by sex and age group, offer a complementary and statistically more powerful outcome measure. In South Korea, Huh et al. formally derived and validated a sex and age specific continuous severity score using nationally representative KNHANES data, demonstrating its strong predictive validity for future cardiovascular disease and type 2 diabetes [13]. By preserving continuous variance, this approach enhances statistical power, eliminates artificial cutoff bias, and tracks progressive cardiometabolic changes across diverse demographic subgroups. Although continuous severity scores have gained wide acceptance in cardiometabolic epidemiology, their application to dietary electrolyte balance remains unexplored.

Using pooled data from the 2022–2024 Korea National Health and Nutrition Examination Survey (KNHANES IX), this study addresses two primary objectives. First, the independent association of a higher dietary Na/K ratio with the risk of hypertension and metabolic syndrome among South Korean adults is examined, with outcomes assessed using standard binary criteria alongside a validated continuous MetS severity score (H1). Second, the potential moderating role of dietary fiber intake on this association is evaluated, under the hypothesis that higher recall-based fiber intake attenuates the adverse association of a high Na/K ratio with these outcomes (H2).

## 2. Materials and Methods

### 2.1. Study Design and Data Source

This cross-sectional study utilized data from KNHANES, a nationally representative, population-based survey administered annually by the Korea Disease Control and Prevention Agency (KDCA). To establish an adequate sample size for subgroup and interaction analyses, data across three consecutive survey cycles (KNHANES IX: 2022, 2023, and 2024) were pooled into a single analytic dataset. The survey employs a stratified, multistage probability cluster sampling design based on geographic area, sex, and age group, ensuring the representativeness of the non-institutionalized South Korean civilian population. KNHANES comprises three core components: a health interview, a health examination, and a nutrition survey. The health interview collects information on sociodemographic characteristics, health conditions, medication use, smoking, and alcohol consumption. The health examination includes standardized anthropometric, blood pressure, and laboratory assessments, whereas the nutrition survey captures dietary intake data obtained through a single 24 h dietary recall.

### 2.2. Study Population

This 2022–2024 survey cycles were pooled to increase the available sample size for the prespecified analyses of the dietary sodium-to-potassium (Na/K) ratio, metabolic syndrome, and the Na/K ratio × dietary fiber interaction. The analysis was restricted to adults aged 20–59 years because the metabolic syndrome severity score equations applied in this study were developed and validated for this age range [13]. Following the application of predefined eligibility and exclusion criteria, the final unweighted analytic sample comprised 8354 participants. According to the pooled KNHANES weights, these participants represented a weighted population of 27,293,126 adults aged 20–59 years, comprising 14,255,970 men and 13,037,156 women.

For the pooled analysis, the KNHANES-recommended combined health examination and nutrition survey weight was divided by three to account for pooling across three survey cycles, in accordance with the KDCA guidance for pooled KNHANES data. All estimates and statistical analyses incorporated the KNHANES stratification variables, primary sampling units, and pooled survey weights.

To minimize confounding and measurement error pregnant or breastfeeding women, individuals missing data on any MetS component or dietary sodium, potassium, or fiber intake, and those reporting implausible daily total energy intake (<500 kcal/day or >5000 kcal/day) were excluded. Participants with physician-diagnosed kidney failure or an estimated glomerular filtration rate (eGFR) below 60 mL/min/1.73 m^2^ were also excluded. The participant selection process and detailed exclusions are presented in Figure 1.

### 2.3. Exposure Variables: Dietary Na/K Ratio

References to sodium, potassium, and fiber “intake” or “consumption” refer to values obtained from a single 24 h dietary recall and reflect the amounts reported on the day the recall was conducted, rather than steady-state dietary exposure. To facilitate comparison across both clinical and nutritional epidemiology literature, the dietary Na/K ratio in two complementary forms:

- Mass-based Na/K ratio: Calculated as the ratio of daily sodium intake (mg/day) to daily potassium intake (mg/day).
- Molar Na/K ratio: Calculated by converting sodium and potassium intakes to molar units (mmol/day) to account for the differing atomic weights of the two elements.

All analyses modeled both metrics as continuous variables.

### 2.4. Outcome Variables

Hypertension was defined as systolic blood pressure ≥130 mmHg, diastolic blood pressure ≥ 85 mmHg, or current use of antihypertensive medication. In accordance with standard KNHANES protocol, trained examiners recorded blood pressure under standardized conditions, with the mean of the second and third consecutive measurements used for analysis.

Metabolic syndrome was defined using Adult Treatment Panel III (ATP-III) criteria adapted for Korean and Asian populations, requiring the presence of at least three of five components [14]:

- Waist circumference ≥ 90 cm in men or ≥85 cm in women;
- Fasting plasma glucose ≥ 100 mg/dL or current antidiabetic medication use;
- Blood pressure ≥ 130/85 mmHg or current antihypertensive medication use;
- Triglycerides ≥ 150 mg/dL;
- HDL cholesterol < 40 mg/dL in men or <50 mg/dL in women, or current antidyslipidemic medication use.

Medication use was ascertained from KNHANES health interview items regarding current treatment for hypertension, diabetes, and dyslipidemia.

Beyond the binary MetS diagnosis, each participant’s continuous, sex- and age-specific MetS severity score was calculated using pre-established equations and beta coefficients obtained directly from the validated study by Huh et al. [13]. Confirmatory factor analysis (CFA) was not re-estimated in the current study sample. Applying the fixed, pre-validated regression coefficients maintains a standardized outcome measure across KNHANES cycles, ensuring direct comparability with prior literature and avoiding dataset specific weight fluctuations that would occur if CFA were re-estimated. These established coefficients were applied across sex and age subgroups (20 to 39 and 40 to 59 years), incorporating waist circumference, systolic blood pressure, fasting plasma glucose, HDL cholesterol, and natural log-transformed triglycerides:

MetS severity score = β_0_ + β_1_(FPG) + β_2_(SBP) + β_3_(WC) + β_4_(HDL) + β_5_(ln[TG])

This continuous score was evaluated as a complementary outcome measure in all primary analyses. To ensure full reproducibility, the exact age- and sex-specific standardized coefficients and equations used to derive the continuous MetS severity score for participants aged 20–39 and 40–59 years are provided in Supplementary Table S1.

### 2.5. Moderator Variable: Dietary Fiber Intake

Total dietary fiber intake (g/day) was calculated from the same 24 h dietary recall used for sodium and potassium estimates. For sensitivity analyses, an energy-adjusted fiber density metric (g/1000 kcal) was additionally evaluated to test whether findings differed between absolute and relative fiber metrics.

### 2.6. Covariates

Multivariable models were adjusted for age, sex, body mass index, current smoking status, alcohol consumption frequency, and total daily energy intake (kcal/day). All covariates were extracted from the health interview, physical examination, and nutrition survey components of KNHANES. Body mass index was included to capture overall adiposity independent of the abdominal adiposity reflected in waist circumference, one of the five components used to define metabolic syndrome and its continuous severity score. Because BMI and waist circumference are only moderately correlated, BMI was retained as a covariate rather than an outcome-embedded measure.

### 2.7. Statistical Analysis

All analyses accounted for the complex sampling design of KNHANES, including stratification, primary sampling units, and sample weights. The 2022, 2023, and 2024 KNHANES cycles were pooled into a single analytic dataset. In accordance with KDCA guidance for pooled KNHANES data, the combined health examination and nutrition survey weight for each cycle was divided by three before analysis. This adjustment prevents the pooled three-year sample from representing the sum of the population estimates for the individual survey cycles. All descriptive statistics, regression models, and hypothesis tests were conducted using survey-weighted procedures implemented in R (version 4.5.3; R Foundation for Statistical Computing, Vienna, Austria; accessed on 24 June 2026) using the survey, gtsummary, and cardx packages. Descriptive characteristics were summarized by sex. Continuous variables are presented as weighted means with standard errors, whereas categorical variables are presented as weighted percentages; unweighted participant counts are also reported. Between-sex comparisons were performed using design-based Kruskal–Wallis tests for continuous variables and Rao–Scott second-order-corrected Pearson’s chi-square tests for categorical variables.

Primary Association Models (H1): To test whether a higher Na/K ratio is independently associated with increased hypertension and metabolic syndrome risk, survey-weighted multivariable regression models were fitted for each outcome. Logistic regression using a quasibinomial family accounted for overdispersion under survey weighting for binary hypertension and MetS outcomes, while linear regression evaluated the continuous MetS severity score. All models were adjusted for age, sex, BMI, smoking status, alcohol consumption, and energy intake. Odds ratios and beta coefficients with 95% confidence intervals were derived from model coefficients using t-distribution-based confidence intervals appropriate for the residual degrees of freedom of the survey design.

Moderation Models (H2): To test whether dietary fiber intake moderates the association between the Na/K ratio and hypertension and metabolic syndrome risk, the Na/K ratio and fiber intake were mean-centered. A multiplicative interaction term representing the Na/K ratio multiplied by fiber intake was added to each of the three outcome models alongside the standard covariate set. Statistical significance of the interaction term at a *p*-value below 0.05 indicated moderation. When a significant interaction occurred, simple slope analysis evaluated the association of the Na/K ratio with the outcome at one standard deviation below the mean (−1 SD), at the mean, and at one standard deviation above the mean (+1 SD) of fiber intake. The Johnson-Neyman technique further identified the range of fiber intake across which the association between the Na/K ratio and MetS severity remained statistically significant, based on the model’s coefficient variance-covariance matrix implemented in R.

Sensitivity Analyses: To evaluate the robustness of significant interaction findings, two sensitivity analyses were conducted. First, variance inflation factors (VIF), including generalized VIF (GVIF) for the interaction term, considering a VIF above 5 as indicative of potential concern. Second, moderation models were re-estimated using an energy-adjusted fiber density metric (g fiber per 1000 kcal) in place of absolute fiber intake to evaluate whether findings depended on the specific fiber metric selected.

Both mass-based and molar Na/K ratios were calculated to facilitate comparison across both clinical and nutritional epidemiological literature. Because molar ratios represent a deterministic mathematical rescaling of mass measurements, these dual metrics serve to provide standardized reporting units rather than an independent sensitivity analysis. Statistical significance was set at a two-sided *p* < 0.05 for all analyses.

## 3. Results

### 3.1. Participant Characteristics

The final unweighted analytic sample comprised 8354 participants, representing a weighted population of 27,293,126 adults aged 20–59 years (Table 1).

Significantly less favorable cardiometabolic profiles were observed in men across nearly every measured parameter, alongside a substantially higher Na/K ratio compared with women (all *p* < 0.001). In line with this risk profile, hypertension prevalence (21.4% vs. 11.9%) and metabolic syndrome prevalence (32.4% vs. 12.7%) were roughly two- to three-fold higher in men than in women (both *p* < 0.001), underscoring the established sex disparity in cardiometabolic burden among South Korean adults.

### 3.2. Associations of the Na/K Ratio with Hypertension and Metabolic Syndrome (H1)

After adjusting for age, sex, BMI, current smoking status, alcohol consumption frequency, and total energy intake the mass-based dietary Na/K ratio was independently and positively associated with all three examined outcomes in multivariable regression models (Table 2).

Each one-unit increase in the mass-based Na/K ratio was associated with 14.4% higher odds of hypertension, 20.4% higher odds of metabolic syndrome, and a significant corresponding increase in the continuous, age- and sex-specific MetS severity score. The magnitude of association appeared larger for composite metabolic syndrome than for hypertension alone.

Across all three models, established cardiometabolic covariates, particularly age and BMI, demonstrated expected strong positive associations. Results remained directionally and statistically consistent regardless of whether the Na/K ratio was expressed on a mass or molar basis. These findings support H1.

### 3.3. Moderation Analysis of Dietary Fiber Intake (H2)

To evaluate whether dietary fiber intake moderates the association between the dietary Na/K ratio and cardiometabolic outcomes, survey-weighted multivariable models incorporating multiplicative interaction terms were estimated. For hypertension (binary), higher mass-based and molar Na/K ratios remained independently associated with increased odds. However, neither the main effect of dietary fiber intake nor the interaction terms reached statistical significance. In contrast, for metabolic syndrome (binary), statistically significant positive interactions were observed between the Na/K ratio and dietary fiber intake in both mass-based models. For the continuous MetS severity score, dietary fiber intake significantly moderated the association with both mass-based and molar Na/K ratios (Table 3).

As illustrated in Figure 2, the predicted probabilities of MetS across dietary fiber strata demonstrate an absolute risk interaction between the Na/K ratio and fiber intake. At low-to-moderate Na/K ratios, higher dietary fiber intake aligns with lower predicted probabilities of MetS. However, as the dietary Na/K ratio increases, the risk trajectory for the high-fiber subgroup (75th percentile) steepens substantially compared to the low-fiber subgroup (25th percentile). At elevated Na/K ratios, high fiber intake fails to confer protection against MetS risk, visually reinforcing the synergistic multiplicative interaction (OR = 1.02, *p* < 0.001) on an absolute risk scale.

To clarify the nature of this interaction, simple-slope analysis of the continuous MetS severity score model was performed. At low dietary fiber intake (−1 SD), the association between the Na/K ratio and MetS severity was weak and non-significant (β = 0.05, *p* = 0.11). In contrast, at mean (β = 0.12, *p* < 0.001) and high (+1 SD; β = 0.19, *p* < 0.001) levels of fiber intake, the association became progressively stronger and statistically significant. This pattern was further evaluated across the observed range of fiber intake using a Johnson–Neyman probe which indicated that the association between the Na/K ratio and MetS severity remained statistically significant across nearly the entire distribution of fiber intake, losing significance only near its lowest tail (Figure 3).

Rather than attenuating the association, higher dietary fiber intake demonstrated a positive statistical interaction with the Na/K ratio on metabolic syndrome severity, indicating that the magnitude of the association varies across levels of fiber consumption. This interaction was specific to the composite metabolic syndrome outcome and its continuous severity index, without extending to hypertension alone. Consequently, H2 was not supported in the hypothesized direction.

To rule out shared variance among the Na/K ratio, fiber intake, and total energy intake as an explanation for the observed interaction, we evaluated variance inflation factors (VIF) in the continuous MetS severity interaction model. All VIF values were well below standard thresholds of concern: Na/K ratio (1.17), fiber intake (1.80), age (1.33), sex (1.47), BMI (1.28), smoking (1.14), alcohol (1.12), energy intake (1.57), and the interaction term (1.14). The absence of meaningful multicollinearity indicates that the observed interaction reflects a genuine statistical association rather than a collinearity artifact.

To determine whether interaction direction and significance were sensitive to fiber operationalization, moderation models were re-estimated by substituting energy-adjusted fiber density (fiber_1000 kcal, g per 1000 kcal) for absolute fiber intake (g/day) (Table 4).

To ensure that findings were not artifacts of ratio formulation or shared variance from a single 24 h recall, sensitivity models were fitted with separate sodium and potassium intakes. In joint main-effect models, sodium was positively associated (β = 0.0012, *p* = 0.008) and potassium was inversely associated β = −0.0009, *p* = 0.014) with continuous MetS severity. In joint interaction models, dietary fiber density significantly moderated the effect of sodium (β = 0.0011, *p* = 0.005) but not potassium (*p* = 0.412), confirming that the observed Na/K ratio interaction is primarily driven by sodium dynamics.

The interaction remained significant and positive for the continuous MetS severity score, demonstrating that this finding is robust to how fiber intake is operationalized. When expressing fiber as an energy-adjusted density rather than an absolute amount, the interaction for binary MetS attenuated to non-significance. This pattern suggests that the unexpected moderation effect depends more on outcome dichotomization than on the specific fiber metric selected. The continuous MetS severity score may thus represent a more sensitive and stable outcome measure for detecting this subtle moderation effects.

## 4. Discussion

In this study representing a weighted population of 27.3 million South Korean adults from the pooled 2022–2024 KNHANES cycles, the independent association between the dietary Na/K ratio and hypertension alongside metabolic syndrome was evaluated (H1), together with the potential moderating role of dietary fiber intake (H2).

In support of H1, a higher Na/K ratio was independently associated with increased odds of hypertension and metabolic syndrome, as well as a higher continuous, sex- and age-specific metabolic syndrome severity score after adjustment for established cardiometabolic covariates. Contrary to H2, dietary fiber intake did not attenuate this relationship. Instead, fiber intake demonstrated a statistically significant positive interaction with the Na/K ratio for metabolic syndrome outcomes in cross-sectional analysis. This statistical interaction remained robust across sensitivity analyses evaluating multicollinearity and alternative fiber metrics, for the continuous severity outcome.

### 4.1. The Na/K Ratio and Cardiometabolic Risk (H1)

The independent, positive association observed between the Na/K ratio and both hypertension and metabolic syndrome aligns with a substantial body of international and specific to the Korean population [9,12]. This body of research links sodium excess and relative potassium deficiency directly to elevated blood pressure and adverse metabolic profiles. The Na/K ratio is increasingly considered a more informative dietary marker than sodium intake alone, as potassium is thought to counteract several pressor mechanisms of sodium, including renal sodium retention, sympathetic nervous system activation, and vascular smooth muscle tone [15]. Consequently, the ratio may capture the net physiological burden of these two cations better than either nutrient evaluated in isolation.

The association with metabolic syndrome (OR = 1.204) was numerically and statistically stronger than that with hypertension alone (OR = 1.144), a pattern mirrored by the continuous MetS severity score. This difference may reflect the entanglement of the Na/K ratio with a broader constellation of dietary and metabolic risk factors. In South Korea, elevated sodium consumption frequently co-occurs with reliance on processed, salted, and fermented foods. Independent of their sodium content, these foods may also introduce excess refined carbohydrates and overall caloric load [16]. This interpretation aligns with recent evidence indicating that the Na/K ratio partly reflects overall dietary quality rather than sodium-potassium physiology alone [17]. However, the present cross-sectional design cannot fully resolve this distinction.

The association of the Na/K ratio with cardiometabolic risk was consistent across three distinct outcome operationalizations: a binary hypertension diagnosis, a binary composite MetS diagnosis, and a continuous validated MetS severity score. This consistency strengthens confidence that the observed association is not an artifact of a specific outcome definition or statistical approach. These findings support the growing consensus that dietary Na/K ratio represents an important modifiable indicator associated with cardiometabolic health in the South Korean adult population [18].

### 4.2. Statistical Interaction of Dietary Fiber (H2)

A central finding of this study is that a statistically significant interaction was observed between dietary fiber intake and the dietary Na/K ratio in relation to metabolic syndrome outcomes. Crucially, this statistical moderation should not be interpreted as indicating that dietary fiber is an inherently harmful or adverse dietary component. This result runs counter to the a priori hypothesis, which was grounded in the physiological rationale that fiber might offset metabolic harm from a sodium-heavy, potassium-poor diet [10,11]. Several non-mutually exclusive explanations merit consideration.

From a clinical magnitude perspective, evaluating this interaction on an absolute risk scale reveals a substantial divergence in predicted MetS prevalence. At balanced Na/K levels (centered Na/K = −1), high fiber intake (75th percentile) is associated with the lowest absolute MetS probability (28%), compared to 34% in the low-fiber group (25th percentile). However, at elevated Na/K ratios (centered Na/K = +6), predicted MetS probability escalates to 85% among high-fiber consumers compared to 55% among low-fiber consumers—a 57 percentage point absolute risk increase versus 21 percentage points, respectively. This demonstrates that while fiber intake offers baseline protection under favorable electrolyte conditions, high reported fiber consumption fails to mitigate, and on an absolute scale accentuates, the risk trajectory driven by an unfavorable Na/K ratio.

Individuals diagnosed with or at elevated risk for metabolic syndrome, hypertension, diabetes, or dyslipidemia frequently receive dietary counseling from healthcare providers emphasizing increased fiber intake as standard risk reduction [19]. Furthermore, reverse causality may play a key role: individuals aware of their elevated cardiometabolic risk or diagnosed metabolic conditions may intentionally increase their dietary fiber consumption as a health-improving behavior, turning high fiber intake into a proxy for higher underlying baseline risk rather than a causal driver. The cross-sectional design cannot rule out this form of reverse causation, representing an important limitation.

Disaggregating the Na/K ratio into its constituent electrolytes revealed that the observed moderation by fiber intake is specifically attributable to sodium rather than potassium. Because sodium, potassium, and fiber were all estimated from a single 24 h dietary recall, shared measurement error across dietary items must be considered. However, the distinct, directional interaction observed exclusively with sodium supports a true physiological interaction rather than systematic dietary recall artifact.

Within the KNHANES dietary recall framework, high total fiber intake may co-occur with dietary patterns that are not uniformly favorable. The traditional Korean diet relies heavily on fiber-containing vegetable side dishes (banchan), many of which contribute substantially to sodium intake, most prominently kimchi alongside other salted, fermented, or seasoned vegetables [20,21]. Participants with higher recall-based fiber intake in this population may have drawn that fiber disproportionately from these high-sodium preparations. Rather than acting as a biologically harmful exposure, high fiber intake in this population may serve as a contextual marker for specific dietary patterns in which fiber-rich plant foods are prepared with substantial amounts of added sodium [4,22]. However, because specific food sources and overall dietary patterns were not directly evaluated in the present analysis, this food matrix explanation remains a plausible hypothesis requiring direct verification in future studies. This interpretation aligns with the finding that the interaction was significant for the absolute fiber metric but attenuated to non-significance for the binary MetS outcome when fiber was expressed as energy-adjusted density. This shift suggests that absolute food quantity, rather than fiber’s relative dietary contribution of fiber, may partly explain the observed pattern.

In the present dataset, the dietary fiber was assessed as total dietary fiber without distinguishing soluble from insoluble fiber fractions. Physiological mechanisms supporting a protective buffering role are most strongly documented for soluble fiber, particularly regarding its impact on postprandial glucose and lipid metabolism through short-chain fatty acid production and altered gut transit [23,24]. Consequently, the observation of a null or unexpected interaction when fiber is measured in aggregate does not necessarily contradict a protective hypothesis specific to soluble fiber. Future investigation incorporating granular fiber-type data would be valuable to clarify this distinction.

Multicollinearity does not account for the observed statistical interaction. In the full model, variance inflation factors (VIFs) for the Na/K ratio, fiber intake, and their interaction term were all below 1.8, well within conventional thresholds of concern. The interaction remained robust across both absolute and energy-adjusted fiber metrics for the continuous MetS severity outcome. However, it was attenuated to non-significance for the binary MetS outcome when using the energy-adjusted metric. This pattern suggests that continuous outcome measures detect this interaction more reliably than dichotomized parameters, consistent with the established statistical principle that dichotomization reduces statistical power to detect moderation [25]. Furthermore, as demonstrated in the revised Johnson–Neyman plot featuring the embedded data distribution (rug/density plot), the conditional effect of the Na/K ratio on MetS severity is supported by the vast majority of the study population. The interaction remains statistically significant across the central distribution of dietary fiber intake, confirming that the observed moderation reflects robust information across the main body of data rather than an artifact driven by sparse observations at the extreme tails of the moderator range.

Several notable strengths characterize the present study. First, data were drawn from three consecutive years (2022–2024) of nationally representative KNHANES cycles, utilizing complex survey design parameters (stratification, clustering, and combined survey weights) to yield population-representative estimates. Second, alongside conventional binary diagnostic criteria a validated, Korean-population-specific continuous metabolic syndrome severity score was utilized. Derived via confirmatory factor analysis and stratified by sex and age, this continuous outcome measure enables the detection of subtle dose–response and moderation patterns those binary outcomes alone may obscure.

Furthermore, evaluating the Na/K ratio using both mass-based and molar metrics provided standardized results directly comparable across clinical and epidemiological literature, although these represent mathematical rescaling rather than independent sensitivity analyses. Formal sensitivity analyses were also conducted to address potential multicollinearity and alternative exposure operationalization. Because such rigorous testing is not universally reported in comparable literature, these analytical steps substantially reinforce the internal validity of the moderation findings.

Limitations: Several limitations of this study warrant consideration. First and most importantly, the cross-sectional design precludes causal inference and cannot rule out reverse causation, or unmeasured dietary confounding, particularly regarding the observed statistical interaction with fiber intake. Second, dietary intake data in KNHANES relied on a single 24 h recall, or unmeasured dietary confounding, particularly regarding the observed statistical interaction with fiber intake. Furthermore, because no statistical adjustment for usual intake was applied, these data do not reflect long-term habitual dietary exposure. Although such measurement error typically attenuates true associations toward the null, rendering the observed effect estimates conservative, all findings must be interpreted strictly as associations with reported day-of-recall intake rather than stable dietary exposure. Third, the fiber variable does not distinguish between soluble from insoluble fiber fractions, limiting mechanistic interpretations. Fourth, the metabolic syndrome severity score equations were derived and validated in an earlier cohort (KNHANES 2010–2015) and are methodologically restricted to individuals aged 20 to 59 years [13]. Excluding adults aged 60 years and older, who present with a higher baseline prevalence of cardiometabolic risk factors and distinct dietary patterns, introduces potential selection bias and limits the generalizability of these findings to older population groups. Fifth, detailed decomposition into major food sources or overall dietary patterns was not conducted in this analysis. Consequently, whether high fiber intake directly captures sodium-dense plant preparations or broader health-seeking dietary shifts cannot be definitively established. Finally, despite adjustment for a substantial set of cardiometabolic covariates, residual confounding from unmeasured factors (e.g., physical activity intensity, socioeconomic status) cannot be fully excluded.

## 5. Conclusions

Based on pooled 2022–2024 KNHANES data, updated, nationally representative evidence regarding dietary electrolyte balance and cardiometabolic health among South Korean adults was established within a survey-weighted analytical framework. A higher dietary Na/K ratio was independently associated with increased odds of hypertension, metabolic syndrome, and greater overall metabolic severity.

Furthermore, dietary fiber intake significantly modified the association between the Na/K ratio and metabolic syndrome outcomes. However, this moderation pattern should be interpreted with caution; high dietary fiber intake likely functions as a contextual marker for traditional, sodium-dense plant food matrices or past health-seeking behavior rather than an adverse biological driver. Public health strategies in South Korea should therefore prioritize optimizing the overall dietary Na/K ratio rather than focusing exclusively on single-nutrient modifications in isolation.

Future research utilizing longitudinal designs will be necessary to establish temporal precedence and clarify causality. Incorporating pattern-based nutritional analyses, such as cluster or principal component analysis of major food groups, will be essential to disentangle specific nutrient effects from co-occurring sodium-rich dietary sources.

## Figures and Tables

**Figure 1:**
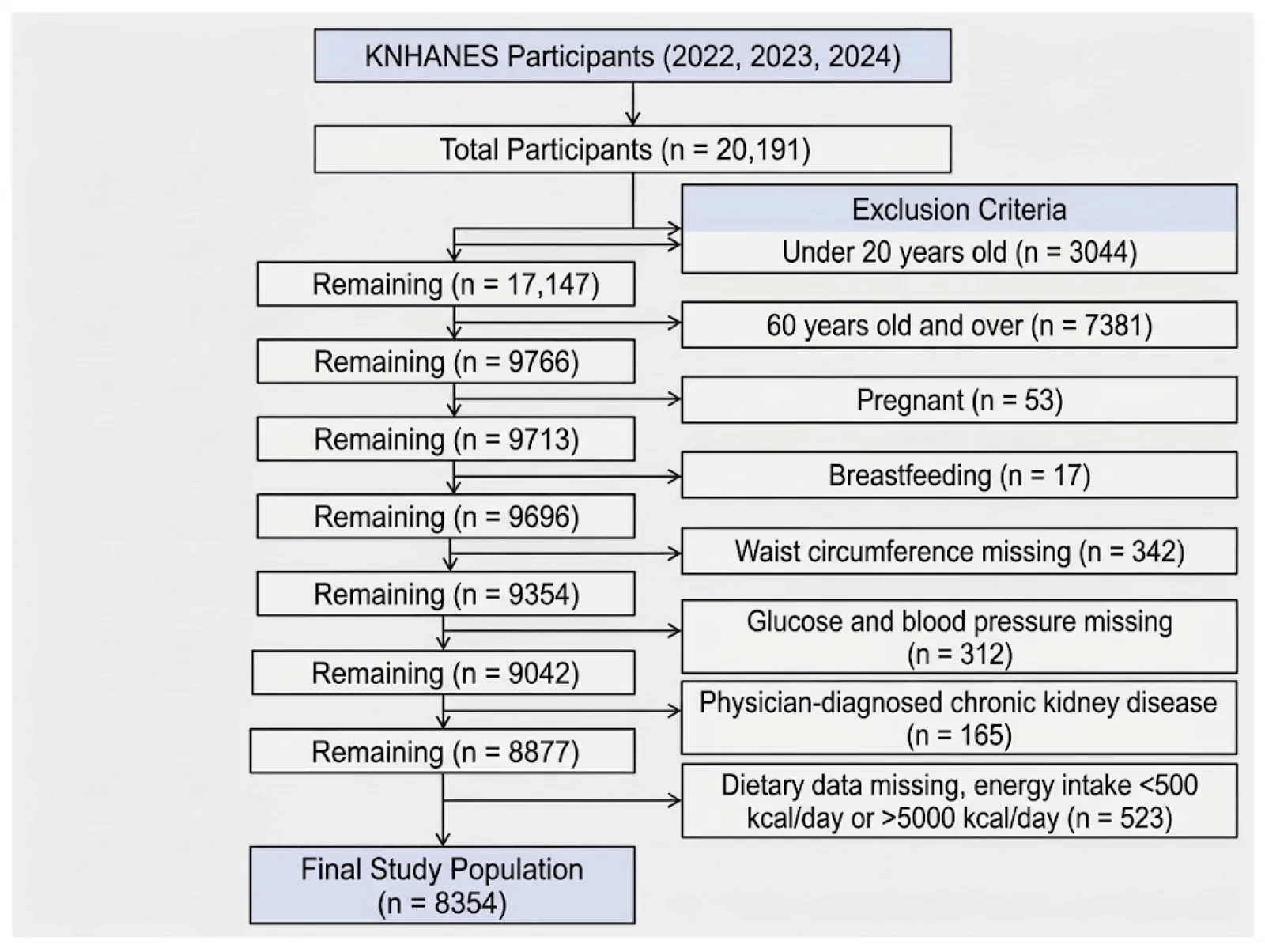
Flow diagram of participant selection from the combined KNHANES IX (2022–2024) cycles.

**Figure 2:**
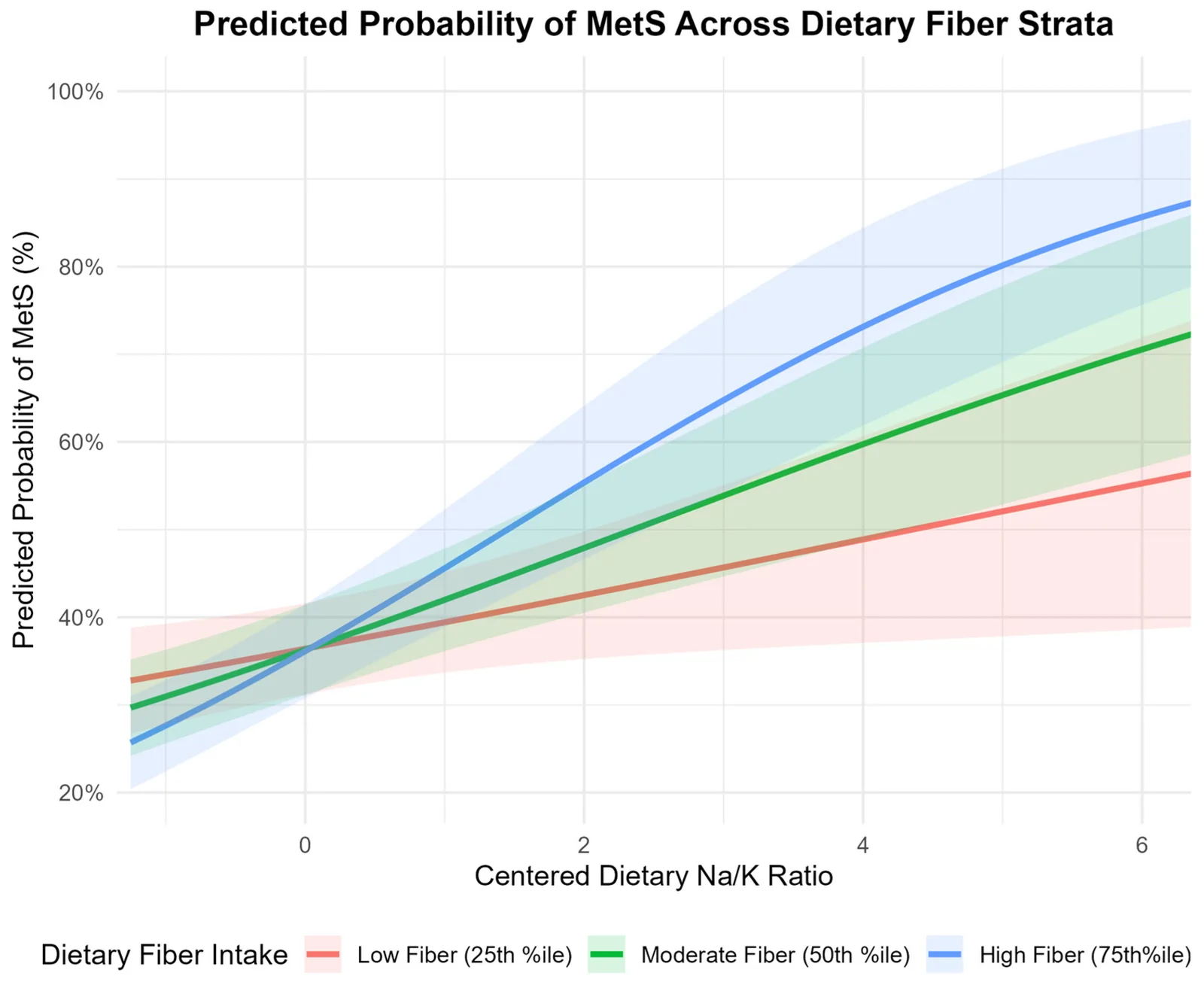
Moderation effect of dietary fiber intake on the association between centered dietary Na/K ratio and the predicted probability of Metabolic Syndrome (MetS). Lines represent predicted probabilities based on simple slope analyses at the 25th percentile (Low Fiber, red), 50th percentile (Moderate Fiber, green), and 75th percentile (High Fiber, blue) of dietary fiber intake. Shaded areas represent 95% confidence intervals.

**Figure 3:**
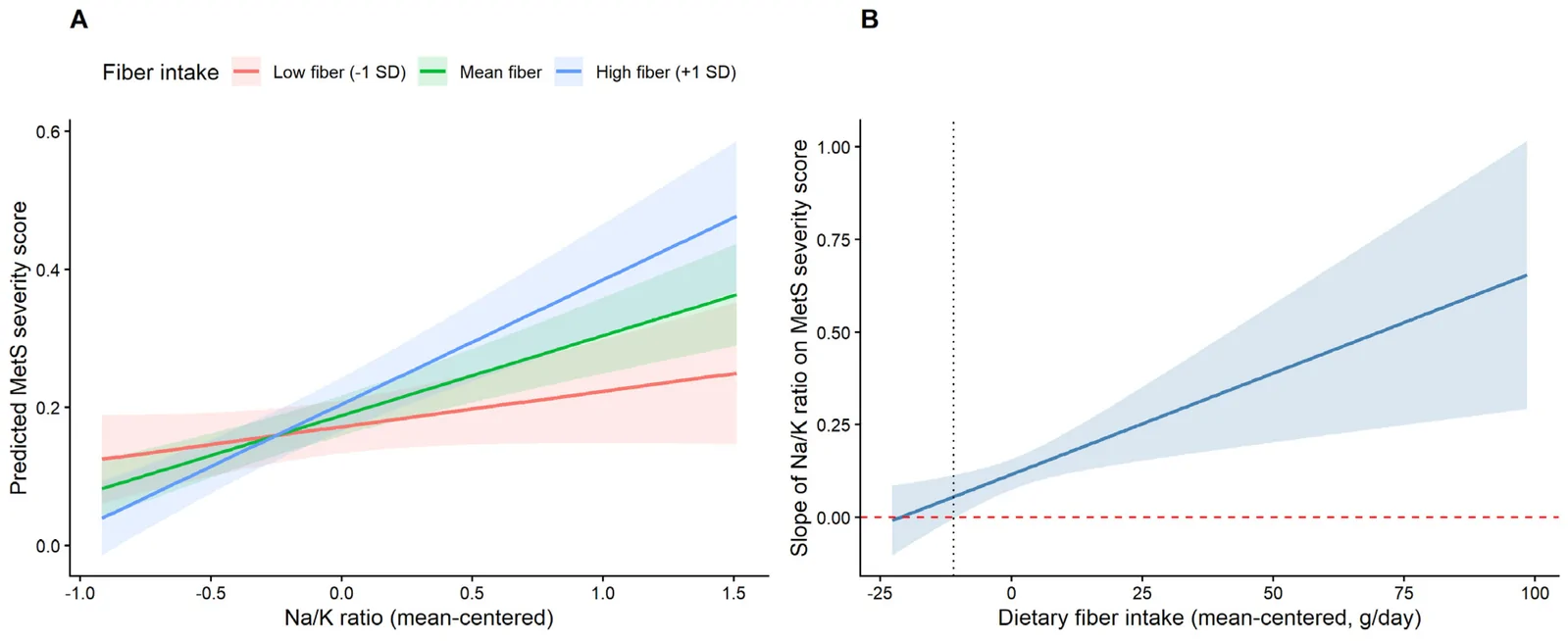
Moderating effect of dietary fiber intake on the association between the Na/K ratio and metabolic syndrome severity score. (**A**) Predicted MetS severity score across the observed range of the mean-centered Na/K ratio (mass-based), plotted separately at low (−1 SD), mean, and high (+1 SD) levels of mean-centered dietary fiber intake, derived from the survey-weighted linear regression model adjusted for age, sex, BMI, smoking status, alcohol consumption, and total daily energy intake. Shaded bands represent 95% confidence intervals. (**B**) Johnson–Neyman plot illustrating the conditional slope of the Na/K ratio on MetS severity score as a continuous function of mean-centered dietary fiber intake (g/day). The shaded region represents the 95% confidence interval of the conditional slope; the horizontal dashed red line indicates a slope of zero; the vertical dotted vertical line marks the Johnson–Neyman significance threshold, below which the association between the Na/K ratio and MetS severity is no longer statistically significant (*p* ≥ 0.05).

**Table 1:** Weighted demographic, anthropometric, biochemical, and dietary characteristics of the study population aged 20–59 years, by sex (KNHANES IX, 2022–2024).

Variable	Overall (Unweighted n = 8354; Weighted N = 27,293,126)	Men (Unweighted n = 3630; Weighted N = 14,255,970)	Women (Unweighted n = 4724; Weighted N = 13,037,156)	*p*-Value
Age (years)	40.63 (0.18)	40.27 (0.24)	41.02 (0.19)	0.005
Waist circumference (cm)	83.04 (0.15)	88.45 (0.19)	77.14 (0.17)	<0.001
BMI (kg/m^2^)	24.23 (0.05)	25.35 (0.07)	23.02 (0.07)	<0.001
Systolic BP (mmHg)	115.34 (0.19)	119.58 (0.25)	110.72 (0.21)	<0.001
Diastolic BP (mmHg)	74.45 (0.15)	77.13 (0.20)	71.52 (0.16)	<0.001
Fasting plasma glucose (mg/dL)	97.36 (0.26)	100.16 (0.40)	94.30 (0.30)	<0.001
Triglycerides (mg/dL)	130.72 (1.37)	158.49 (2.40)	100.46 (1.13)	<0.001
HDL cholesterol (mg/dL)	58.11 (0.21)	51.88 (0.25)	64.90 (0.28)	<0.001
Sodium intake (mg/day)	3312.87 (22.36)	3865.80 (31.45)	2710.42 (23.66)	<0.001
Potassium intake (mg/day)	2635.90 (15.32)	2857.64 (20.93)	2394.29 (18.15)	<0.001
Na/K ratio (mass-based)	1.33 (0.009)	1.44 (0.013)	1.22 (0.010)	<0.001
Na/K ratio (molar)	2.27 (0.015)	2.45 (0.022)	2.08 (0.018)	<0.001
Dietary fiber intake (g/day)	22.63 (0.15)	24.54 (0.21)	20.55 (0.18)	<0.001
Energy intake (kcal/day)	1914.52 (9.77)	2189.20 (14.15)	1615.24 (9.83)	<0.001
Hypertension (%)	16.8%	21.4%	11.9%	<0.001
Metabolic syndrome (%)	23.0%	32.4%	12.7%	<0.001
Antihypertensive medication (%)	9.9%	12.0%	7.6%	<0.001
Antidiabetic medication (%)	4.5%	5.8%	3.1%	<0.001
Antidyslipidemic medication (%)	9.6%	10.2%	9.0%	0.067

Values are weighted mean (SE) for continuous variables and weighted % for categorical variables, with unweighted sample counts (n) reported in the column headers. *p*-values are derived from design-based Kruskal–Wallis tests for continuous variables and Rao-Scott-adjusted Pearson’s chi-square tests for categorical variables.

**Table 2:** Survey-weighted multivariable associations of dietary Na/K ratio and dietary fiber intake with hypertension, metabolic syndrome, and continuous MetS severity score among South Korean adults aged 20–59 years (KNHANES IX, 2022–2024).

Variable	Outcome	O.R./β	95% CI	*p*-Value
Dietary Na/K ratio	Hypertension	O.R. = 1.144	1.023–1.279	0.018
	Metabolic syndrome	O.R. = 1.204	1.076–1.347	0.001
	MetS severity score	β = 0.088	0.034–0.141	0.001
Dietary Fiber Intake	Hypertension	O.R. = 1.003	0.993–1.012	0.581
	Metabolic syndrome	O.R. = 1.010	1.002–1.018	0.013
	MetS severity score	β = 0.0058	0.0023–0.0094	0.001

O.R. = Odds ratio; β = Unstandardized Regression Coefficient; CI = Confidence Interval.

**Table 3:** Survey-weighted moderation analysis of dietary fiber intake on the association between the dietary Na/K ratio and cardiometabolic outcomes among South Korean adults aged 20–59 years (KNHANES IX, 2022–2024).

Outcome and Model	Model Type	Variable/Term	Effect Type	Estimate (95% CI)	*p*-Value
Hypertension (Binary)					
Mass-based Na/K Model	Logistic (Quasibinomial)	Na/K ratio (mass-based)	Main Effect	OR = 1.144 (1.023 to 1.279)	0.018
		Dietary fiber intake (g/day)	Main Effect	OR = 1.003 (0.993 to 1.012)	0.581
		Na/K ratio × Fiber intake	Interaction	OR = 1.003 (0.995 to 1.012)	0.581
Molar Na/K Model	Logistic (Quasibinomial)	Na/K ratio (molar)	Main Effect	OR = 1.085 (1.013 to 1.162)	0.019
		Dietary fiber intake (g/day)	Main Effect	OR = 1.003 (0.993 to 1.012)	0.581
		Na/K ratio × Fiber intake	Interaction	OR = 1.002 (0.997 to 1.008)	0.557
Metabolic Syndrome (Binary)					
Mass-based Na/K Model	Logistic (Quasibinomial)	Na/K ratio (mass-based)	Main Effect	OR = 1.204 (1.076 to 1.347)	0.001
		Dietary fiber intake (g/day)	Main Effect	OR = 1.010 (1.002 to 1.018)	0.013
		Na/K ratio × Fiber intake	Interaction	OR = 1.010 (1.002 to 1.018)	0.013
Molar Na/K Model	Logistic (Quasibinomial)	Na/K ratio (molar)	Main Effect	OR = 1.121 (1.045 to 1.203)	0.001
		Dietary fiber intake (g/day)	Main Effect	OR = 1.010 (1.002 to 1.018)	0.013
		Na/K ratio × Fiber intake	Interaction	OR = 1.006 (1.001 to 1.011)	0.013
MetS Severity Score (Continuous)					
Mass-based Na/K Model	Linear (Gaussian)	Na/K ratio (mass-based)	Main Effect	β = 0.088 (0.034 to 0.141)	0.001
		Dietary fiber intake (g/day)	Main Effect	β = 0.006 (0.002 to 0.009)	0.001
		Na/K ratio × Fiber intake	Interaction	β = 0.006 (0.002 to 0.009)	0.001
Molar Na/K Model	Linear (Gaussian)	Na/K ratio (molar)	Main Effect	β = 0.052 (0.021 to 0.083)	0.001
		Dietary fiber intake (g/day)	Main Effect	β = 0.006 (0.002 to 0.009)	0.001
		Na/K ratio × Fiber intake	Interaction	β = 0.003 (0.001 to 0.005)	0.001

OR = Odds Ratio; β = Unstandardized Regression Coefficient; CI = Confidence Interval. All models account for the complex survey design of KNHANES IX (2022–2024) using primary sampling units, strata, and pooled survey weights adjusted for 3-year pooling (n = 8354; weighted N = 27,293,126).

**Table 4:** Sensitivity analysis of the Na/K ratio × dietary fiber interaction on metabolic syndrome outcomes, using energy-adjusted fiber density (g per 1000 kcal).

Outcome	Interaction β	Approx. 95% CI †	*p*-Value
Metabolic syndrome	0.0013	−0.0017 to 0.0196	0.889
MetS severity score	0.0095	0.0067 to 0.0242	0.003

† Calculated using survey design-based t-distribution confidence intervals corresponding to the residual degrees of freedom of the complex sampling design (df = 62).

## Data Availability

The dataset analyzed during the current study originates from the Korea National Health and Nutrition Examination Survey (KNHANES) and can be accessed via the official Korea Disease Control and Prevention Agency (KDCA) portal at https://knhanes.kdca.go.kr (accessed on 1 June 2026).

## Supplementary Materials

The following supporting information can be downloaded at: https://www.mdpi.com/article/10.3390/healthcare14193145/s1, Table S1. Sex- and age-specific sample-weighted regression coefficients (β) for constructing the continuous Metabolic Syndrome Severity Score (KNHANES IX, 2022–2024).
