# Supplementary material for: When Fiber Does Not Protect: Evaluating Statistical Interaction Between Dietary Fiber, Sodium-to-Potassium Ratio, and Metabolic Syndrome in South Korean Adults
Source: Healthcare (Basel). 2026 Sep 22;14(19):3145. doi: 10.3390/healthcare14193145 (PMC13664825; doi:10.3390/healthcare14193145)
Supplement: Supplementary file 1 [file healthcare-14-03145-s001.zip › healthcare-4552640-supplementary.pdf]

Supplementary Table S1. Sex- and age-specific sample-weighted regression coefficients (β) for constructing the continuous Metabolic Syndrome Severity Score (KNHANES IX, 2022–2024).

**Parameter / Term**	**Men (20–39 yrs)**	**Women (20–39 yrs)**	**Men (40–59 yrs)**	**Women (40–59 yrs)**
**Intercept (β_0)**	-5.421	-4.892	-6.115	-5.654
**Fasting Glucose (β_1, mg/dL)**	0.0125	0.0118	0.0142	0.0131
**Systolic BP (β_2, mmHg)**	0.0182	0.0165	0.0201	0.0189
**Waist Circumference (β_3, cm)**	0.0410	0.0435	0.0385	0.0412
**HDL-Cholesterol (β_4, mg/dL)**	-0.0215	-0.0198	-0.0231	-0.0208
**ln[Triglycerides] ($\beta_{5,}$ mg/dL)**	0.4210	0.3980	0.4520	0.4150

Fasting plasma glucose (FPG), systolic blood pressure (SBP), waist circumference (WC), and high-density lipoprotein cholesterol (HDL-C) are entered in their raw clinical units. Triglycerides (TG) are entered after natural logarithmic transformation (ln[TG]).
